# 3D Printing and Bioprinting to Model Bone Cancer: The Role of Materials and Nanoscale Cues in Directing Cell Behavior

**DOI:** 10.3390/cancers13164065

**Published:** 2021-08-12

**Authors:** Tiziana Fischetti, Gemma Di Pompo, Nicola Baldini, Sofia Avnet, Gabriela Graziani

**Affiliations:** 1 Department of Biomedical and Neuromotor Sciences, Alma Mater Studiorum-Università di Bologna, 40138 Bologna, Italy; tiziana.fischetti2@unibo.it (T.F.); sofia.avnet3@unibo.it (S.A.); 2 Biomedical Science and Technologies Lab, IRCCS Istituto Ortopedico Rizzoli, 40136 Bologna, Italy; gemma.dipompo@ior.it; 3 Laboratory of NanoBiotechnology, IRCCS Istituto Ortopedico Rizzoli, 40136 Bologna, Italy

**Keywords:** 3D printing, 3D bioprinting, bone cancer, calcium phosphates, bone model, orthopedics

## Abstract

**Simple Summary:**

Three-dimensional bioprinting is a promising tool for the study of cancer development and progression in bone, as it permits modeling the complexity of the microenvironment and cell-to-cell interactions. To this aim, an ideal model should combine a proper structure design, biomaterials selection, and the cellular counterpart. In this review, 3D-bioprinted bone systems obtained by different bioinks, and strategies, are discussed, aimed at mimicking the bone cancer microenvironment. The main challenges and unmet needs to reach perfect biomimicry are highlighted.

**Abstract:**

Bone cancer, both primary and metastatic, is characterized by a low survival rate. Currently, available models lack in mimicking the complexity of bone, of cancer, and of their microenvironment, leading to poor predictivity. Three-dimensional technologies can help address this need, by developing predictive models that can recapitulate the conditions for cancer development and progression. Among the existing tools to obtain suitable 3D models of bone cancer, 3D printing and bioprinting appear very promising, as they enable combining cells, biomolecules, and biomaterials into organized and complex structures that can reproduce the main characteristic of bone. The challenge is to recapitulate a bone-like microenvironment for analysis of stromal–cancer cell interactions and biological mechanics leading to tumor progression. In this review, existing approaches to obtain in vitro 3D-printed and -bioprinted bone models are discussed, with a focus on the role of biomaterials selection in determining the behavior of the models and its degree of customization. To obtain a reliable 3D bone model, the evaluation of different polymeric matrices and the inclusion of ceramic fillers is of paramount importance, as they help reproduce the behavior of both normal and cancer cells in the bone microenvironment. Open challenges and future perspectives are discussed to solve existing shortcomings and to pave the way for potential development strategies.

## 1. Introduction

Bone cancer can arise as primary (sarcomas) or metastatic lesions. Bone sarcomas, including osteosarcoma and Ewing’s sarcoma, are highly aggressive tumors, mainly affecting pediatric patients and young adults [1]. Although the advent of chemotherapy has considerably prolonged life expectancy, bone sarcomas are still associated with a 5-year survival rate of approximately 50–60% due to their resistant or recurrent nature, thus representing a leading cause of cancer-related death in young people [2]. Bone is also the third most common metastatic site in patients affected by breast, prostate, lung, and renal carcinoma. Bone metastatic progression leads to 90% of death from cancer [3,4] and is associated with a significant decrease in the 5-year survival rates [5,6,7,8,9] and severe morbidities, including pain, fracture, hypercalcemia, and spinal cord compression [7,10,11]. At this stage, the disease is usually considered incurable, and treatment is only palliative, consisting of pain-relieving medication, radiation therapy, and surgery [12,13,14].

To date, the availability of tissue samples of bone cancers has been limited by the difficulty of reaching the bone site. Furthermore, adjuvant therapy is often administered prior to surgery. Because the therapy has some cytotoxicity, it may alter the integrity of DNA, RNA, and proteins, or interfere with the metabolic and the proliferation activities of cells of the tumor microenvironment, all prior to tissue sampling. As a consequence, this may alter the native characteristics and behavior of cells, thus affecting the relative molecular and morpho-histological analyses [15]. Consequently, the study of the biological mechanisms underlying bone tumors and the development of successful strategies for their treatment and prevention are very difficult. In this context, preclinical modeling of bone microenvironment appears to be a crucial and promising challenge. For decades, evaluation of cancer cell proliferation, migration, invasion, and drug response has relied on two-dimensional (2D) in vitro cell culture systems. However, such models fail to mimic the spatial, biochemical, and mechanical complexity of the native three-dimensional (3D) tumor microenvironment, that is, tissue architecture, severely limiting their interpretation in the study of primary and secondary bone cancer [2]. In vivo models overcome these drawbacks by mimicking the physiological context of tumor growth and progression, thus being more predictive of drug response compared to 2D cultures. Nevertheless, studies on animals are limited by ethical concerns, species-specific differences, and high costs. In addition, non-spontaneous cancer models, such as syngeneic, xenografts, or orthotropic models, also fail to recapitulate the paracrine circuits by which the bone niche modulates bone cancer progression and response to treatments. This is because they often develop too rapidly, which impedes the establishment of the natural interactions between cancer cells and stromal cells that occur in vivo [16,17,18].

Three-dimensional in vitro models can help to bridge the gap between preclinical in vitro and in vivo models, as they are highly reproducible, affordable, support the use of human cells, and can recapitulate the key features of the bone tumor niche, such as 3D cell–cell and cell–extracellular matrix (ECM) interactions, therefore facilitating mechanistic and drug response studies [19,20]. To this aim, to date, various 3D techniques have been developed, including multicellular spheroids, microfluidic chips, cell patterning techniques, and 3D printing [21]. Thanks to the combination of these advanced technologies with different types of biomaterials, versatile approaches can be obtained to develop 3D cellular constructs that can recapitulate the tumor microenvironment complexity.

To study bone tumors, the model shall mimic as closely as possible the composition and properties of the native bone tissue, merging biological and materials science-related requirements. Bone, however, is a complex tissue, composed of a mineral and an organic phase, and by cells, all arranged in a highly hierarchical structure [22,23,24]. Therefore, the model shall also possess a certain degree of complexity and fulfill several requirements. Among these, (i) excellent biocompatibility, (ii) suitable surface properties, (iii) adequate mechanical properties, (iv) a porous structure that can allow cell colonization and vascularization, and (v) tailored degradability are those identified as mandatory in the literature [22,25,26].

In this scenario, 3D bioprinting offers new perspectives, as it allows easily producing porous structures having finely tunable architecture, mechanical properties, and composition [27,28]. In these models, surface characteristics (morphology and roughness) can be modulated from the macro to the submicrometric scale by tailoring the model shape and porosity. By loading the models with nanoscale materials (nanocoatings or nanoparticles), features at the nanoscale can also be obtained while creating a hybrid organic/inorganic composition. The use of inorganic micro- or nano-fillers permits increasing printing fidelity, and further modulates degradability, stability, mechanical properties, and interactions with host cells [29,30,31,32,33]. However, in 3D printing, cells can be seeded onto the scaffolds but cannot be incorporated in the fibers, hindering the study of cell–cell and cell–ECM interactions [34,35]. To address these limitations, 3D bioprinting can be used to create complex in vitro cancer and bone models that can replicate different aspects of the 3D tumor microenvironment [36,37,38] and may be useful for understanding tumor heterogeneity and identify those mechanisms responsible for tumor progression and resistance to therapy [36,39]. More in detail, 3D bioprinting permits: (i) printing multiple cell types, including cancer and normal cells associated with the tumor microenvironment [40]; (ii) enabling the formation of vessel-like structures that are crucial to study the metastatic process and to assess anti-cancer drug delivery and responses [41]; (iii) modulating the composition of the exogenous ECM for what regards both the inorganic matrix and the various growth factors or signaling molecules [36,38]. This is important as it permits simulating the loose or dense connective tissues surrounding the cells in the tumor microenvironment, thus providing a reliable re-establishment of the existing crosstalk between cancer cells and neighboring matrices [21,38]. Consequently, both 3D printing and bioprinting, combined with nanoscale materials, appear promising to simulate specific properties of the bone tissue and study cancer.

In summary, biofabrication technologies, combined with specifically engineered materials, enable the printing of biomimetic 3D structures with detailed morphological features, from the millimeter to nanometer range [42], and fine control over the spatial positioning of cells during the bioprinting process [43,44,45,46]. For this reason, 3D printing and bioprinting have opened new routes, overcoming the limitations of overly simplified traditional 2D cultures in mimicking heterogeneity and complexity of both native [40] and tumor tissues [36,38].

In this review, we will focus on new trends in the development and manufacturing of 3D osteomimetic scaffolds obtained through additive manufacturing techniques for the study of osteosarcoma and bone metastases. The state of the art and advances regarding novel organic/composite bioinks, inorganic fillers, and new strategies for biomimetic scaffold development are systematically reviewed, and the main challenges, opportunities, and future perspectives are highlighted.

## 2. The Bone Microenvironment: Key Features for 3D Modeling of Bone Cancer

Bone sarcomas and bone metastases share the same environment and niche. Once established in bone, they interact with normal resident cells and with physical stimuli, including mechanical stress and hypoxia [47]. When cancer cells colonize the bone compartment, they start to proliferate, invade, and disrupt the normal bone matrix and acquire an osteomimetic phenotype, thereby interacting with bone cells [48,49,50,51,52].

### 2.1. Interaction with Normal Cells

**Figure 1:**
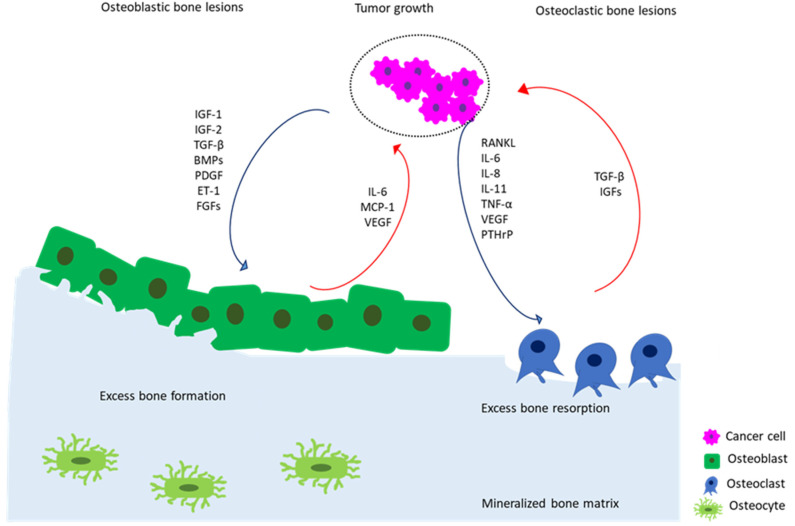
Schematic depiction of vicious cycle of bone metastasis involving the complex mutual interactions between tumor cells and bone cells in osteoblastic and osteoclastic bone lesions. Tumor cells secrete pro-osteoblastic (i.e., IGF-1 and -2, TGF-β, BMPs, PDGF, ET-1, and FGFs) or pro-osteoclastic (i.e., RANKL, IL-6, IL-8, IL-11, TNF-α, VEGF, and PTHrP) mediators (blue arrows) that induce bone formation or bone resorption, respectively. In turn, in osteoblastic lesions, osteoblasts produce pro-tumor growth factors (i.e., IL-6, MCP-1, and VEGF) that further stimulate the growth of cancer cells (red arrows). In osteolytic lesions, osteoclast-mediated bone resorption induced by cancer cells triggers the release of pro-tumor growth factors (i.e., IGFs and TGF-β) from the bone matrix, thus fueling the vicious cycle of cancer growth (red arrows).

The cellular environment of the bone tumor niche is comprised of complex and dynamic interactions between tumor and normal resident cells, including osteoblasts, osteoclasts, endothelial, immune, and hematopoietic cells, all of which are implicated in the pathogenesis of bone cancers [48]. Invading cancer cells strongly affect the activity of osteoblasts and osteoclasts (bone-forming cells and bone-resorbing cells, respectively), thereby disrupting the physiological balance of bone remodeling. Therefore, abnormal bone tissue formation and/or dysregulated bone resorption may occur, resulting in osteoblastic or osteolytic lesions [53] (Figure 1).

In osteoblastic lesions (i.e., osteosarcoma and prostate cancer metastases), tumor-derived growth factors (i.e., insulin growth factors (IGF)-1 and -2, transforming growth factor-beta (TGF-β), bone morphogenetic proteins (BMPs), platelet-derived growth factor (PDGF), endothelin-1 (ET-1), and fibroblasts growth factors (FGFs)) stimulate the differentiation and bone-forming activity of osteoblasts. In turn, osteoblasts produce growth factors that further stimulate tumor growth, such as interleukin-6 (IL-6), monocyte chemoattractant protein 1 (MCP-1), or vascular endothelial growth factor (VEGF) [54].

In cancer-induced osteolytic bone disease, such as breast cancer metastases, cancer cells secrete a variety of cytokines and growth factors, including receptor activator of nuclear factor kappa-Β ligand (RANKL), IL-6, IL-8, interleukin 11 (IL-11), tumor-necrosis factor-alpha (TNF-α), vascular endothelial growth factor (VEGF), and parathyroid hormone-related protein (PTHrP) [32], which directly or indirectly stimulate osteoclasts to resorb bone. The process of bone resorption, in turn, causes the release of additional growth factors from the matrix, such as IGFs and TGF-β, that can also favor cancer progression [48]. Among the paracrine pro-osteoclastogenic factors produced by cancer cells, RANKL, PTHrP, IL-11, and VEGF have particular relevance [55]. Furthermore, in the acidic tumor microenvironment, tumor-activated osteoblasts or mesenchymal stromal cells (MSCs) can secrete inflammatory cytokines, such as IL-6 and IL-8, that, in turn, further boost bone disruption and tumor progression [56].

However, only taking into consideration the interactions of osteoblasts and osteoclasts with tumor cells is an oversimplification. In fact, as for lung [57,58], brain [59,60,61], colon [62,63], and breast cancer [41,59], tumor-induced vasculature is a critical factor for the survival and proliferation of cancer cells in bone. During the uncontrolled growth of the tumor, oxygen and nutrient deprivation strongly stimulate the pro-angiogenic activity of tumors cells, inducing the secretion of angiogenic growth factors and cytokines, such VEGF and IL-8, into the surrounding extracellular microenvironment. This dysregulated signaling pathway activates the adjacent endothelial cells and perivascular cells, causing the recruitment of new blood vessels, which further support the growth of the tumor [64,65].

In summary, modeling the complex interactions between resident bone cells and tumor cells by considering all the different cellular players is crucial to recapitulate the molecular and cellular mechanisms of bone cancers in vitro.

### 2.2. Interaction with the Bone Matrix

Upon spreading to the skeleton, cancer cells not only interact with bone cells but also with the ECM. The latter is a dynamic structure including both organic and inorganic components that contribute to the functioning of the musculoskeletal system [66]. Although it is mainly composed of type I collagen (≈90%) [67], bone ECM also comprises several non-collagenous proteins, including fibronectin and lysyl oxidase (LOX). The inorganic phase of bone (≈75–80 wt.%), instead, is constituted by biogenic apatite (BA) nanocrystals (also called biological hydroxyapatite, or bone apatite), which allow for mineral exchange.

Biophysical properties of bone ECM are crucial in determining cell phenotype and behavior, both in normal and cancer cells. In fact, (i) the cell–matrix interactions in bone can affect cell migration, proliferation, survival, and remodeling [68], and (ii) ECM cues can promote tumor growth and decrease the response to therapeutics [69]. In addition, (iii) ECM stiffness can modulate the stemness and the expression of epithelial–mesenchymal transition (EMT) markers in osteosarcoma cells [70]. Finally, (iv) several studies have shown that hydroxyapatite (HA, Ca_10_(PO_4_)_6_(OH)_2_) can affect the behavior of normal and cancer cells [29,71], thereby validating the importance of including the ceramic counterpart in 3D in vitro tumor models [72]. In conclusion, reproducing a composition as similar as possible to that of native tissue is crucial to investigate how cues provided by the bone matrix can modulate cancer cell phenotype, growth, and chemoresistance.

## 3. Additive Manufacturing for Printing Bone-like Tissues

In bone oncology, the development of 3D models by additive manufacturing is still at its early stages, and several key issues are yet to be investigated. On the contrary, in orthopedics, biomimetic 3D constructs have been largely applied for the regeneration and repair of native bone tissue [44,73,74,75,76,77,78]. The knowledge acquired in this field can thus be advantageously translated to create models and solutions for the study of cancer cell development and progression in bone.

### 3.1. Printing Versus Bioprinting

Additive manufacturing is a very promising and versatile technique that allows the development of 3D constructs through a layer-by-layer process in which various biomaterials can be combined and possibly mixed with different cell types and/or growth factors [79,80]. Additive manufacturing is referred to as 3D bioprinting or printing, respectively, depending on whether cells are included in the printing process. Embedding the cells into the ink has some advantages and drawbacks that depend on the tissue to model and its specific characteristic. Models manufactured by 3D printing require the cell to be seeded on the surface of 3D constructs, so the technique is also known as “indirect bioprinting”. These models permit high freedom in the choice of the materials to be printed, so they better mimic mechanical and structural properties of bone, as well as its degradation profile [80]. The so-obtained models may have long-term stability and can be inserted into bioreactors. However, seeding cells onto the 3D constructs does not allow for homogeneous cell dispersion and scaffold colonization [81,82], thus partially allowing for the simulation of cell–cell and cell–ECM interactions.

Instead, 3D bioprinting permits the creation of a defined distribution of cells and/or biomolecules inside the ink and hence across the fibers of the whole scaffold [26,45,83], which is necessary to mimic the biological complexity of cancer [44,84]. In addition, the use of natural hydrogels, having high water content, guarantees high biocompatibility and the possibility to tune the chemical and physical characteristics of the ink (including viscosity, crosslinking, and concentration, all determining shear stress) by selecting the appropriate polymeric matrix. As a consequence, they permit creating a microenvironment compatible with the medium-term survival of the cells embedded in the ink [85]. However, different from 3D-printed scaffolds, 3D-bioprinted constructs show limited mechanical properties and lower stability, so the models are not suitable for applications that require mimicking mechanical stress or are for use in bioreactors. In bone 3D printing, the most used synthetic polymers are polycaprolactone (PCL) [86,87,88,89,90], polyethylene glycol diacrylate (PEGDA) [31,78], and polylactic acid (PLA) [41], because of their mechanical strength, structural properties, and biocompatibility, both in vitro and in vivo. These polymers, largely used for 3D printing, have high rigidity and slow degradation rate [28,87,91]. However, they need to be processed in aggressive conditions (dilution in acid/toxic solvents and/or at high temperature) and cannot incorporate cell media [28,87]. Hence, cell loading of the polymeric fibers is impeded, which makes them unsuitable for bioprinting.

An optimal ink for 3D bioprinting shall fulfill the needs of high printing fidelity, shape maintenance, cell viability, and function [79]. These outcomes are affected by several parameters, both depending on the ink, such as: (i) chemical composition, including polymer concentration and molecular weight [85,92]; (ii) viscosity (hydrogels with shear-thinning characteristics are desired [92,93]), and cell density (suitable cell concentration in the order of 10^6^ cells/mL, corresponding to approximately 5% of the total bioink volume [44]). The chemical composition of the ink is the main parameter regulating cells response, as described in Section 3.3.2. However, the ink’s physical characteristics (i.e., polymer concentration, viscosity, and crosslinking mechanism) also have importance in determining cells viability in the short, medium, and long term [79,85,94,95].

**Figure 2:**
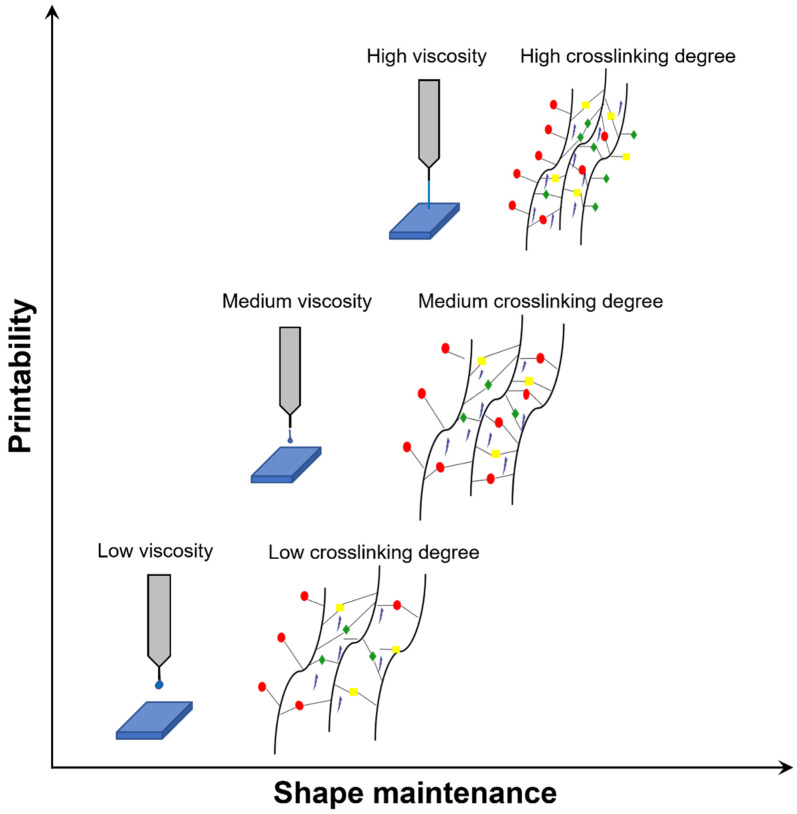
Trend of printability and shape maintenance depending on bioink viscosity (related to ink concentration) and crosslinking degree. Bioink type and the reported parameters need evaluation for each 3D bioprinting experiment. Generally, low/intermediate values of these parameters are preferable to guarantee cell viability.

More in detail: (i) high polymer concentrations permit obtaining dense polymer chains, resulting in increased mechanical properties and stiffness. However, increased density causes a lower diffusion rate of the nutrients, which reduces cell viability and proliferation [79,96]. (ii) High viscosity increases printability and shape fidelity but also shear stress, which negatively impacts cells viability [79,95,97]. Similarly, (iii) a high degree of crosslinking increases mechanical properties but decreases cell viability [79,85,95]. Consequently, the bioink choice is not trivial, as it depends on multiple and opposing parameters (Figure 2). For an extensive overview, see [68,69].

Based on these considerations, in 3D bone bioprinting, natural polymers are preferred, such as alginate [73,98,99,100,101], gelatin [98,100,102], and gelatin methacrylate (GelMA) [103,104,105,106], silk fibroin [107,108], chitosan [75,109,110], hyaluronic acid [76,111], fibrin [86,112], and collagen [31,109,113]. Considering biomimicry, collagen and its denatured counterpart, gelatin, are the most promising, although alginate is often preferred due to the easiness of printing. The selection of the hydrogel is of paramount importance, as it determines printability (shape fidelity and printing conditions), cell survival, and cell–cell interactions. Furthermore, for the development of bone tumor models, it must be considered that 70–75% of bone is composed of a mineralized phase [24,67,114,115], BA, which is a multi-substituted nanocrystalline HA. This phase has a strong influence on tumor and bone cell behavior (viability, morphology, differentiation, etc.) and promotes cancer cells’ proliferation and release of IL-8 [29,71]. For this reason, a mineralized model can be more suitable than a polymeric one for the study of bone cancer. As already reported, there are two ways to include a mineralized fraction in 3D-printed and bioprinted scaffold: directly by adding micro/nanoparticles in the osteomimetic ink [30,31,116,117] or by grafting on the surface as a coating [117,118], the latter being more diffused in 3D printing technology [119].

**Figure 3:**
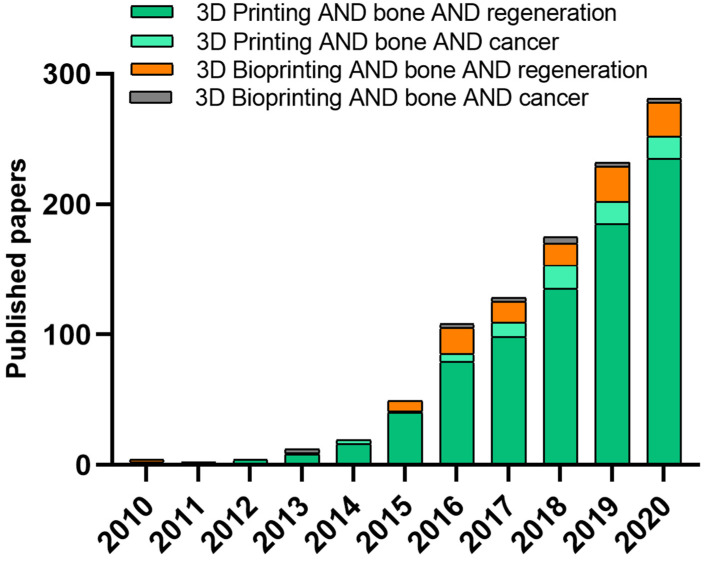
Comparison of uses of 3D printing and 3D bioprinting approaches over time (based on Web of Science, type of document was article, keywords for “3D printing” AND “bone” AND “regeneration”, “3D printing” AND “bone” AND “cancer”, “3D bioprinting” AND “bone” AND “regeneration” and “3D bioprinting” AND “bone” AND “cancer”).

Although a wide and increasing number of studies investigate 3D printing of biomimetic inks for applications in bone tissue regeneration, to date, only a few focus on 3D printing for bone tumor modeling, and an even lower number takes into account bioprinted constructs for tumor modeling (Figure 3). Moreover, not all the performed studies consider the inclusion of a biomimetic or non-biomimetic ceramic phase. Detailed examples and results will be reported in the next paragraphs. However, the increasing trend of research studies on these topics clearly shows their relevance.

### 3.2. Bioactive and Bioinert Bioceramic Fillers in Bioprinting

Bioceramic can be used to functionalize both natural and synthetic polymers and tune mechanical properties, viscosity, and/or stability of the ink, as well as its architecture and biological properties [74,120,121,122]. In particular, the addition of bioceramic fillers, independently of their composition, provides increased mechanical properties [121,123,124], partially overcoming the intrinsic limitations of the hydrogels. At the same time, they affect the rheological characteristics of the inks and permit higher shape fidelity and stability over time [31,125]. To these aims, both bioinert and bioactive compounds can be selected.

However, the use of bioactive compounds (calcium phosphates CaPs and/or bioactive glasses—BG) can provide additional benefits: (i) the release of ions (such as Ca, P, Mg, Na, etc.) that are present in bone, simulating the tissue environment and interacting with healthy and tumor cells; (ii) topographical cues both at the micro- and the nano-scale, that can support cells adhesion to the models surface and directly influence their behavior (for instance, Nano-HA particles/coatings has been reported to direct early differentiation of MSCs [126,127,128]). Increased adhesion, in turn, facilitates seeding, cell spreading, and proper colonization of all parts of the model.

Among the most investigated bioceramic (for a detailed description of different types of bioceramic, see [129,130]), hydroxyapatite [31,75,99] is the most used because of its similarity to the inorganic phase of bone. However, although bone apatite is somehow similar to pure HA, they differ in terms of composition, ion doping, stoichiometry, crystallinity degree, crystal size/morphology, and, consequently, solubility and ions release into the biological medium [131]. Indeed, BA is characterized by low crystallinity and a high solubility and is ion-doped. In BA, carbonate ions substitute for hydroxide and phosphate ions, changing into the formula Ca_10-2x/3_(PO_4_)_6-x_(CO_3_) _x_(OH)_2-x/3_. Besides carbonates, BA contains a significant amount of foreign ions, such as magnesium, fluorine, strontium, silicate, zinc, and manganese, all having specific and significant biological roles [72]. Thus, in comparison to pure or stoichiometric HA, ion-substituted or BA better mimics normal bone tissue [72]. As a demonstration, a large number of studies on bone tissue regeneration have confirmed that BA improves adhesion and proliferation of osteoblast-like cells and osteogenic differentiation of osteoblast precursors, as demonstrated by increased alkaline phosphatase (ALP) and mineralization activity, both in vitro and in vivo [31,116,124,132,133,134,135,136,137,138,139,140,141].

Due to its putative pro-tumorigenic effect [142], HA has also been used to model bone cancers, in particular, osteolytic bone metastases from breast carcinoma [106,116]. Typically, secondary tumor formation in bone is considered a function of bone resorption because the degradation of bone mineral matrix releases bioactive ions and soluble growth factors that, in turn, are critical for the proliferation of both normal and cancer cells [143]. However, insoluble cues inherent to the inorganic component of the bone mineral matrix may also regulate metastatic growth by promoting adhesion, proliferation, and colonization of tumor cells, as highlighted in [116] when comparing a mineralized and a non-mineralized scaffold for breast cancer modeling. In the same study, the presence of HA in the mineralized scaffolds also promoted the release of IL-8 from breast cancer cells that, in turn, exerted pro-tumorigenic and pro-osteolytic effects [71], thereby supporting the vicious cycle of tumor growth and bone resorption. Hence, incorporation of a bone-like mineralized component into engineered cancer models may allow the study of the molecular mechanisms behind HA-induced metastasis in bone or, possibly, the study of HA-promoted drug resistance of cancer cells in bone. In another study on the same type of cancer [29], the effects of HA particles were studied by varying their size, crystallinity, and synthesis route, and assessing their effects on protein adsorption, cancer cells adhesion, growth, and IL-8 secretion. Protein adsorption, cell adhesion, and proliferation increased with decreasing HA crystallinity and crystal size. In contrast, IL-8 secretion reached the highest level in scaffolds with highly crystalline HA [29]. Data obtained by this study are very interesting, and it would be worthy to investigate the same behavior by using other cancer types that are prone to grow or metastasize to bone. However, although a large body of literature is already available on the use of functionalized nanoparticles and their biological role [31,117,124,144,145,146], research on printing of ion-doped CaPs is only in its early infancy, and further development is expected in the next years. Finally, of note, all the studied particles were used at one same scale (e.g., nanoparticles as nanoscale cues to modulate cells colonization in the scaffolds, but also to boost early differentiation and influence morphology), whereas the study of the effects of multi-scale particles is still unexplored.

### 3.3. Bioprinting: Cells and Bioinks

#### 3.3.1. Normal Cells Used in 3D Bioprinting for Mimicking the Bone Microenvironment

The most used cells for 3D bone bioprinting in orthopedics are murine or human MSCs from either bone marrow or adipose tissue [87,110,113,147,148,149,150,151], murine calvarial MC3T3-E1 pre-osteoblast cells [46,68,152], and human fetal osteoblasts [105,147,153]. Printing of osteocytes has also been recently proposed [154]. These cells, when cultured in osteoinductive media or in media added with growth factors (i.e., bone morphogenic protein-2 BMP-2 [76,132,155,156,157,158], FGF-2 [112,159], VEGF [74,147]) and/or other additives (i.e., Ca^++^) [102,107], express osteogenic markers (i.e., ALP, osteonectin (ON), osteopontin (OPN), osteocalcin (OCN)) and markers of late osteocyte phenotype (i.e., podoplanin (PDPN) and sclerostin (SOST)), and are able to mineralize.

#### 3.3.2. Biomimetic Inks

An ideal biomimetic ink for cancer modeling should mimic the structural, physico-chemical, and biological properties of ECM. Indeed, to study the mechanisms underlying tumorigenesis and cancer progression, biomimetic inks should mimic the substrate on which the tumor develops and grows. On the other hand, they should reproduce ECM–cells interactions, thereby allowing the study of the mechanisms occurring in osteolytic or osteoblastic lesions, both in primary and secondary bone tumors.

Silk fibroin and chitosan [75,107,110,120,158,160,161], collagen [113,133,145,150,154] and hyaluronic acid [76,78,116,148,151], chemically modified (i.e., methacrylation reaction) or used in blends [98,105,116,161] are among the most widely natural hydrogels employed to induce bone formation. These polymers have biological and chemical features resembling the organic ECM components of bone native tissue. To best mimic bone ECM, the addition of inorganic counterparts as bioceramic (i.e., bioactive glass [30,31,87,100,156], β-tricalcium phosphate (TCP) [89,130,162], HAs [31,73,75,99,122,149], and nanoclays in powders or micron (nanoparticles) [74,78]) has been fully investigated. Three-dimensional bioprinting is promising in the obtainment of 3D cell-laden constructs based on osteomimetic inks combining polymeric matrix and ceramic fillers. To date, positive results have already been obtained regarding either the process, such as printability and extrudability, and the properties resulting from the obtained constructs, such as mechanical strength and stability maintenance. For instance, increased osteogenic ability and mineralization were obtained by using Laponite nanosilicates [74,78], Poly–Ca^2+^complex (i.e., PolyP mixed with CaCl_2_) [30,98] and β-TCP particles [89,130,162], even in absence of an osteogenic medium. These data offer important insights for the development of biomimetic models, as the same technologies can be adapted to reproduce “synthetic bone” with mineralized fractions. To this regard, the studies carried out for bone regeneration also stress the importance of an accurate selection of the following material characteristics: (i) composition (for instance, differences in biological behavior were assessed between scaffolds doped with different BG, with higher mineralization for borate instead of silicate glasses [30,71,152]); (ii) morphology (particle shape, dimensions, and surface features affect the overall behavior of the scaffold); and (iii) mechanical properties.

Most of the studies focus on HA, as it more closely resembles the composition of bone. HA positively influences mechanical and biological properties of the constructs, including the extent of mineralization and collagen production exerted by host cells [73,75,99,156], besides their osteogenic differentiation [19]. In these terms, HA shows more promising results compared to BG nanoparticles [31]. It has also been observed that results in osteoinduction and mineralization may be affected by the hydrogel combination with HA and by the specific characteristics of HA particles (for instance, carbonated HA nanoparticles show increased solubility and hence bioactivity, compared to the stoichiometric counterpart [75]; see Section 3.2).

#### 3.3.3. Vascularized 3D-Bioprinted Bone-like Constructs

Along with the addition of inorganic fillers, a bioprinted model of bone cancer should incorporate vasculature, as it is essential to mimic both normal and cancer cell behavior. In normal musculoskeletal development and regenerative processes, blood vessels have different functions: (i) providing an efficient transport network for molecules and hematopoietic cells, (ii) nourishing niches for hematopoietic stem cells that reside within the bone marrow, and (iii) supporting bone formation and homeostasis [163]. On the other hand, during cancer progression, tumor-induced vascularization fosters tumor growth and dissemination by providing oxygen and nutrients and by supporting the intravasation and extravasation of cancer cells [164]. Tumor angiogenesis is initiated by environmental stresses, such as hypoxia and acidosis, leading to a disequilibrium in the pro-/anti-angiogenic balance and consequently to the increased expression of pro-angiogenic factors, including hypoxia-induced factor (HIF) and VEGF. Although the formation of a tumor vascular network starts from the existing healthy blood vessels, its expansion may be aided by additional processes, such as vasculogenesis and vascular mimicry [165].

Therefore, the development of 3D-bioprinted bone-like constructs incorporating vasculature is essential to recapitulate and study the multistep process of cancer development in bone, both for primary tumors and metastases. To reproduce the osteogenic and vasculogenic niches of bone in vascularized bone constructs, different approaches have been reported [86,104,118,152,165,166,167,168]. These include different combinations of composite materials (i.e., natural hydrogels blended with rigid polymers and bioceramic fillers) and cell types (i.e., MSCs and human umbilical vein endothelial cell HUVEC). Hence, a functional vascularized bone model should possess: (i) high mechanical stability and durability, (ii) specific biological cues, and (iii) osteoconductive properties, which can be obtained by the combined use of rigid/synthetic polymers, natural hydrogels, and bioceramic fillers, respectively [169]. The fulfillment of these requirements has shown promising results in the expression of osteogenic and angiogenic markers (i.e., Angiopoietin-1 (Ang-1), FGF-2 and VEGF). Furthermore, it has been reported that co-culturing HUVECs and MSCs boosted cell proliferation and vascular network development [166]. Finally, dynamic perfusion culture through a bioreactor system [168] can be beneficial for both bone and vascular regions, as the combination of liquid flux and mechanical cues (e.g., shear stress) enhance osteogenic differentiation, mineralization, and VEGF expression. The here-reported strategies, though lacking in reproducing the complexity in the combination of the vascular and bone region, are promising for the development of the vascular network in 3D bone construction.

## 4. 3D-Bioprinted Models of Bone Cancers

To date, only a few 3D-bioprinted in vitro cancer models have been proposed, and an even lower number has been published on bone sarcomas and metastases (Table 1). Among these, the majority exploits indirect 3D bioprinting, where cells (either tumor cells, bone cells, or co-cultures) are not embedded in the scaffold fibers but seeded onto its surface. These studies focus on: (i) the effects of scaffolds geometry and composition on cancer cells proliferation, (ii) cancer cell chemoresistance compared to 2D cultures, and (iii) the effects of the direct and indirect interplay between stromal and cancer cells.

Regarding bone sarcomas, a few studies have shown the effect of bioceramic fillers on proliferation and mineralization of 3D-bioprinted Saos-2 osteosarcoma cell lines (Section 3.3.2) [30,98]. Notably, although these studies consider SaOS-2 as osteoblast-like cells for bone tissue engineering applications, the obtained results can be directly translated to models for osteosarcoma growth in bone.

Among the different types of carcinomas that metastasize to bone, breast cancer is the most frequent and, hence, the most studied in the field of 3D bioprinting. In particular, breast cancer cells are often co-cultured with stromal cells of the bone microenvironment, such as the MSCs and the osteoblasts [19,78,147,170,171,172,173,174], since they support the key events in breast carcinoma metastasization and progression, including migration and drug resistance [175,176].

More in detail, Holmes et al. [173] used fused deposition modeling-based 3D bioprinting for studying bone colonization by breast cancer cells. Three-dimensional bone scaffolds were obtained by PLA, then modified through carboxyl nanocrystalline HA coatings. Square and hexagon patterns (250 and 150 µm size) were chosen because they mimic the random orientations of ECM in bone. Among the chosen patterns, small hexagonal pores were the ones that allowed the highest proliferation of breast cancer cells. This study confirmed that the nanosurface texturization provided by HA offers a biomimetic and tunable bone model that can effectively simulate bone invasion and colonization by metastatic carcinoma cells [173].

**Table 1:** Summary of reports on 3D bone bioprinting for the development of 3D bone tumor in vitro models.

3D Printing Technology	Materials	Type of Cells	Results	Ref.
Extrusion bioprinter	Alginate, gelatin Overlay with agarose layer and PolyP-Ca^++^ complex (100 µM)	SaOS-2 (5 × 10^5^ cells/mL)	- PolyPCa^2+^ enhanced structure stability - PolyPCa^2+^ metabolic degradation by cells - PolyPCa^2+^ modulator of gene expression in SaOS-2	[98]
Extrusion bioprinter	Alginate, gelatin Addition of PolyP, silica, or biosilica + BG nanoparticles (55 nm)	SaOS-2 (5 × 10^5^ cells/mL)	- Formation of mineral nodules composed of Ca-phosphate, Ca-carbonate	[30]
Fused deposition modeling	PLA, HA coating (wet deposition)	MDA-MB-231, MSCs	- Young’s moduli between 30 and 50 MPa, suitable for biomimetic mechanical cues - Effective adhesion of breast cancer cells on HA-coated scaffolds	[173]
Stereolithography bioprinter	Polyethylene glycol (PEG), PEG-DA nHA 10 wt% (wet deposition) Grain size: width = 25 nm, length = 50–100 nm	MDA-MB-231 (5 × 10^5^ cells/scaffold, MSCs (1.5 × 10^5^ cells/scaffold)	- 3D-printed scaffold retains native characteristics of in vivo tumor - Homogenous dispersion of HA nanoparticles in the scaffold - Larger number of spheroids and enhanced migration when HA was added to the scaffolds	[146]
Stereolithography bioprinter	PEG, PEG-DA nHA 10 wt% (wet deposition) Grain size: width = 25 nm, length = 50–100 nm	MDA-MB-231 (5 × 10^5^ cells/scaffold), Human fetal osteoblasts (hFOBs) (5 × 10^5^ cells/scaffold)	- Homogeneous dispersion of HA within the matrix - nHA-PEG suitable microenvironment for cell attachment and proliferation - Multicellular spheroids similar to natural tumor structure	[170]
Stereolithography bioprinter	GelMA (different concentrations), nHA 10 wt% (wet deposition) Grain size: width = 25 nm, length = 50–100 nm	MSCs or osteoblasts (1 × 10^6^ cells/mL) MDA-MB-231 (1 × 10^6^ cells/mL)	- Uniform porosity and good dispersion of nHA within the scaffolds - GelMA + nHA suitable for studying MSCs/breast cancer and osteoblasts/breast cancer cells in vitro	[147]
Stereolithography bioprinter	GelMA, PEGDA (different concentrations) nHA (different concentrations) (wet deposition) Grain size: width = 25 nm, length = 50–100 nm	MDA-MB-231 Endothelial cells hFOBs (1 × 10^4^ cells/mL)	- Multi-interaction of tri-culture (cancer–vessel–tissue) - Mechanical properties lower than physiological range but suitable for bone cells growth - Vascular environment important for directional migration of cancer cells	[41]

**Figure 4:**
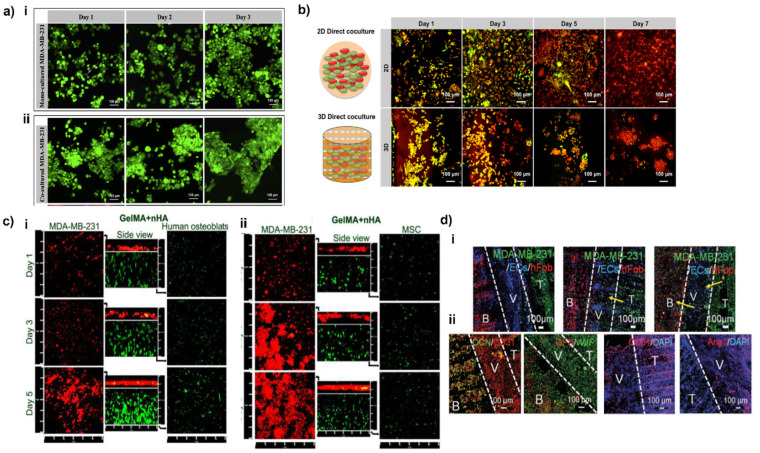
Effect of tumor-healthy cell interactions in co-culture systems and in 3D vs. 2D models. (**a**) Morphology of breast cancer cells cultured alone or with MSCs. (i) Confocal images of MDA-MB-231 alone, and (ii) in co-culture with MSCs; green fluorescence represents Cell Tracker Green™ stained breast cancer cells. Reproduced with the permission of © 2015 Elsevier Inc. All rights reserved [146]. (**b**) Enhanced spheroid formation by direct co-culture of hFOB and MDA-MB-231 cells on the 3D matrix in comparison to monolayer culture. hFOB and MDA-MB-231 were pre-stained with cell tracker green and orange, respectively. Reproduced with the permission of © IOP Publishing. All rights reserved [170]. (**c**) Confocal micrographs of osteoblasts/breast cancer cells (i) and MSCSs/breast cancer cells (ii) co-cultured in the 3D-bioprinted matrix after 1, 3, and 5 days. The middle columns represent the cross-sectional views. Osteoblasts and breast cancer cells were stained by Cell Tracker Green CMFDA dye (green) and Orange CMTMR dye (red), respectively. Reproduced with the permission of © 2016, American Chemical Society [147]. (**d**) Development of MDA-MB-231 cells metastasis and colonization toward bone over 14 d of the culture period. Cell tracker imaging was conducted to monitor the BrCa invasive process, including breast cancer growth, transendothelial migration, and colonization. The yellow arrows indicate the migration of invasive breast cancer cells. (i) Immunofluorescent images of hFOB and MDA-MB-231 cells in a vascular environment with DAPI staining after 14 d of culture. CD31 and vWF staining were used to identify both EC and breast cancer cells. (ii) Osteogenesis of hFOB was characterized by OCN and OPN staining. Combining CD31 and Ang1 was used to distinguish the breast cancer cells and endothelial cells. B: bone tissue, V: vessel, T: tumor tissue. Reproduced with the permission of Wiley-VCH GmbH [41].

Conversely, Zhu et al. used stereolithography-based 3D printing to create 3D bone models with 500 µm and 250 µm square and hexagonal pores and co-culture human MSCs and MDA-MB-231 breast cancer cells on the scaffold. In this study, the authors demonstrated that pattern geometry greatly influences cell proliferation. Small square patterns produced the strongest mitogenic effect. In this study, PEG and PEGDA resins were functionalized by HA nanoparticles and printed. MDA-MB-231 cells cultured on the 3D scaffolds were able to migrate and form distinct and spheroidal 3D structures (Figure 4a(i)), which was not observed in 2D culture. The obtained spheroidal morphology was emphasized when MDA-MB-231 were co-cultured with MSCs, thus showing the effect of the tumor-associated mesenchymal stroma in regulating cancer cell behavior (Figure 4a(ii)). Furthermore, the addition of HA nanoparticles promoted cell–matrix interactions and the formation of MDA-MB-231 larger spheroids compared to the bare 3D matrix. Finally, MDA-MB-231 cultured on the 3D scaffold showed a phenotype more resistant to the anti-cancer drug 5-fluorouracil compared to 2D matrices, possibly due to a reduced drug penetration in the 3D in vitro tumor microenvironment [146]. These findings further corroborate the existence of differences in the drug sensitivity of cancer cells when cultured in 3D instead of 2D models. Indeed, it has been widely demonstrated that 3D models recapitulate cell–matrix interactions and enhanced ECM synthesis, thereby mimicking the in vivo tumor microenvironment. In turn, ECM deposition reduces the penetration of drugs into the tumor mass [145], while 2D monolayered cell cultures are directly exposed to drug treatment.

Further confirmation of the possibility to modulate drug response by a 3D biomimetic environment was shown by Han et al., who demonstrated the ability of a 3D-printed biomimetic model of the bone niche to host metastatic breast cancer cells isolated from patient-derived xenografts (PDX). These models showed a drug response to cisplatin similar to the in vivo model, thus supporting the use of 3D printing for drug testing. This possibility was also confirmed in other types of cancers, such as cervical [177], brain [60,178], lung [179], and bladder [179,180]. The quick rising of novel bioprinted models of several types of cancer for drug screening and personalized medicine approaches, as well as the increasing trend of publications on bone models for oncology (Figure 3), clearly indicates that numerous studies will be published in the upcoming years for bone tumors as well.

In another study, PEG/PEGDA + nHA scaffolds were investigated to assess the interactions between hFOBs and MDA-MB-231 cells. The authors used a stereolithography-based 3D bioprinter to create 3D bone models with square pore patterns and a transwell culture system to evaluate the crosstalk between MDA-MB-231 and osteoblasts. In this system, the two cell populations were physically separated but able to exchange medium and secreted cytokines. This study aimed to recreate the microenvironment of bone metastases and to study the effect of bone-invading breast cancer cells on osteoblast activity, with a specific focus on their effect on cell proliferation, on the synthesis of proteins necessary for bone repair, and on the secretion of inflammatory cytokines, which may stimulate osteoclasts activity and are relevant to breast cancer progression in bone [15,181]. The co-culturing induced a significant effect on cells proliferation, increasing proliferation of MDA-MB-231 cells and decreasing that of hFOBs, respectively. Furthermore, co-culturing MDA-MB-231 and hFOB cells led to an increase in the secretion of IL-8, both by MDA-MB-231 and by hFOBs, up to three-fold higher for hFOBs, when in the presence of MDA-MB-231. Comparing 2D and 3D direct co-culture models, differences were also observed in cancer cell growth. While in 2D models, cancer cells grew in monolayer regardless of hFOBs presence (data acquired at 7 days), in 3D-printed matrices, they arranged in spherical aggregates, forming spheroid-like structures (≈100 µm diameter) even at early culture time points (Figure 4b) [170]. Data reported enlightened the importance of both compositional and morphological cues, alongside the possibility to tune cells response by patterning and adding bio-and nano-bioceramic. These results pave the way for a systematic study of these aspects in combination with 3D printing and bioprinting.

The interactions between cancer and stromal cells were also studied by Zhou et al., who used a 3D stereolithography-based bioprinting technique to fabricate a 3D biomimetic bone matrix able to recreate a bone-like microenvironment. In this study, for the first time, MSCs and osteoblasts were embedded in matrices composed of GelMA and nanocrystalline HA, later seeded with breast cells. The model’s aim was to develop a 3D bioprinting bone tumor model. As for Zhu et al. [170], the addition of cancer cells in the model reduced the proliferation of both osteoblasts (Figure 4c(i)) and MSCs (Figure 4c(ii)), while the macromolecules that these two cell types secrete promote cancer cells growth [147]. Additionally, the secretion of VEGF, a crucial regulator of angiogenesis, was overexpressed in tumor cells in co-cultures with MSCs and osteoblasts, whereas, in the same culture, ALP activity, a marker of osteogenesis, was decreased for both MSCs and osteoblasts.

Finally, a further example of bioprinting for modeling and studying breast cancer and the metastasization process to the bone is the study of Cui et al. [41], who used a 3D stereolithography technology for the creation of a model with three distinct regions: (i) a compartment enriched with breast cancer cells, (ii) a vessel with endothelial cells, and (iii) a zone mimicking micro-vascularized bone. The model was developed to study the metastatic process of carcinoma cells intravasating through the endothelial barrier and then extravasating to the bone region. To create the distinct regions, GelMA and PEGDA were used in different concentrations for cancer and bone matrices, and nHA was added to the latter to simulate the inorganic phase of bone. GelMA was also employed to print the vessel interposed between the bone and the cancer matrix. To foster cell seeding on the printed matrices (hFOBs/endothelial cells and MDA-MB-231 cell on bone and cancer matrices, respectively), 3D-printed matrices were mounted on GelMA, and then the vessel was printed in the central part. It was observed that, over a 7-day culture period, MDA-MB-231 cells migrated to the bone matrix. Migration was even accelerated by the presence of endothelial cells in the central vessel, showing the crucial role of these cells in cancer progression. When MDA-MB-231 cells colonized the bone matrix, hFOBs showed a decreased proliferation, while MDA-MB-231 were strongly stimulated to a mitogenic phenotype possibly by the cytokines secreted by osteoblasts, as observed in [170]. Moreover, the growth of endothelial cells in co-culture was slower than in monoculture, suggesting that the factors secreted by MDA-MB-231 inhibited endothelial cell proliferation (Figure 4d(i)). Furthermore, the upregulation of CD31 angiogenic marker and the downregulation of OPN and OCN (Figure 4d(ii)) confirmed the pro-angiogenic activity and the osteogenic inhibition of cancer cells [41].

Overall, the reported 3D models show accurate and reproducible results in terms of mimicking cancer and stromal cell behavior in the bone metastasis microenvironment, thus representing valuable research tools for bone cancer research.

## 5. Conclusions and Future Perspectives

Bone cancer (sarcoma and metastases) are associated with high mortality and complications rate, so more predictive models are needed to study the progression of the disease and the efficacy of therapy.

Higher predictivity requires a better mimicry of the characteristics of the bone and the tumor microenvironment, and new biomaterials-assisted strategies can help overcome this unmet challenge. Among the new strategies, 3D-printed and bioprinted models can offer new perspectives for mimicking the composition, architecture, and physicomechanical characteristics of bone. In addition, the recent development of multi-material bioprinting systems appears promising to allow the simultaneous deposit of the desired cell types (i.e., stromal cells, immune cells, cancer-associated fibroblasts, and microvascular cells [21,36,44]) and the recapitulation of the cancer microenvironment at different stages of cancer progression. Moreover, preliminary results regarding the inclusion of a vascularization compartment indicate that this approach could significantly improve the biological and physiological relevance of 3D in vitro cancer models.

However, the development of 3D models is still at its early stages, with very few results being published. Although the different strategies and results obtained in bone tissue engineering are a useful benchmark for a deeper understanding and further development of in vitro bone models, many challenges are yet to be addressed:

Increased cell model complexity. The cellular environment of the bone tumor niche is comprised of complex and dynamic interactions between tumor and normal resident cells, but the available studies only consider the interactions of osteoblasts and osteoclasts with tumor cells, which is an oversimplification.

Better mimicry of ECM complexity in terms of composition, stiffness, and complexity of the organic and mineral phases. To this aim, the inclusion of HA in the models appears important, as it dictates normal and tumor cells behavior. At the same time, biomimicry could be further increased, by incorporating ion-substituted or BA in the models, to recapitulate the crystallinity, solubility, and ion-availability of the bone environment.

Increased use of ceramic to merge nanoscale morphological cues and biomimetic composition. This approach allows the model ions naturally present in bone to have an important biological role. Ceramic permits increasing the stability and mechanical properties of the models and guarantees higher adhesion of cells to the scaffold’s surface. Thanks to the tunable (and not yet exploited) properties of CaPs, several parameters of the model can be regulated, including printability, viscosity, and shear stress during printing (depending on particles shape and dimensions, which can be determined by selecting the specific CaP and its ion-doping), and CaPs stability/solubility (depending on CaP type and ion-doping, crystallinity, and specific surface).

For 3D printing, a more detailed study on the strategies for the incorporation of ceramics in the model, for instance, by nanostructured coatings and/or more controlled distribution of the nanoparticles. Coatings obtained by wet synthesis might lack homogeneity and adhesion to the substrate. On the other side, nanoparticles might unevenly distribute and generally remain in the bulk of the fiber, with a few available on the surface for interaction with the surrounding microenvironment.

A combination of 3D printing and bioprinting strategies merges the advantages of the two techniques.

More detailed studies show pores’ shape/size and surface patterning. Available studies show that patterning can improve the characteristics of the model, but a systematic investigation is needed to determine which are the best architectural parameters to be selected.

Vascularization. This aspect is highly neglected, but it influences several parameters that are of paramount importance in tumor progression and drug response, including hypoxia, nutrients and oxygen diffusion, and shear stress.

## Abbreviations

2D	Two-dimensional
3D	Three-dimensional
Ang-1	Angiopoietin-1
ALP	Alkaline phosphatase
BA	Biogenic apatite
BG	Bioactive glass
BMP-2	Bone morphogenic protein-2
CaPs	Calcium phosphates
ET-1	Endothelin-1
EMT	Epithelial–mesenchymal transition
ECM	Extracellular matrix
FGF	Fibroblast growth factor
GelMA	Gelatin methacrylate
HA	Hydroxyapatite
hFOB	Human fetal osteoblasts
HIF	Hypoxia-induced factor
HUVEC	Human umbilical vascular endothelial cells
IGF	Insulin growth factor
IL-6,8,11	Interleukin-6,8,11
LOX	Lysyl oxidase
MSCs	Mesenchymal stromal cells
nHA	Hydroxyapatite nanoparticles
OCN	Osteocalcin
ON	Osteonectin
OPN	Osteopontin
PCL	Polycaprolactone
PDGF	Platelet-derived growth factor
PDPN	Podoplanin
PEG	Polyethylene glycol
PEGDA	Polyethylene glycol diacrylate
PTHrP	Parathyroid hormone-related protein
PLA	Polylactic acid
RANKL	Receptor activator of nuclear factor-kappa Β ligand
SOST	Sclerostin
TCP	Tricalcium phosphate
TGF-β	Transforming growth factor-beta
VEGF	Vascular endothelial growth factor
